# Nanotechnology-Based Drug Delivery to Improve the Therapeutic Benefits of NRF2 Modulators in Cancer Therapy

**DOI:** 10.3390/antiox10050685

**Published:** 2021-04-27

**Authors:** Zerrin Sezgin-Bayindir, Sonia Losada-Barreiro, Carlos Bravo-Díaz, Matej Sova, Julijana Kristl, Luciano Saso

**Affiliations:** 1 Department of Pharmaceutical Technology, Faculty of Pharmacy, Ankara University, 06560 Ankara, Turkey; 2 REQUIMTE-LAQV, Department of Chemistry and Biochemistry, Faculty of Sciences, University of Porto, 4169-007 Porto, Portugal; sonia@uvigo.es; 3 Department of Physical Chemistry, Faculty of Chemistry, University of Vigo, 36200 Vigo, Spain; cbravo@uvigo.es; 4 Department of Pharmaceutical Chemistry, Faculty of Pharmacy, University of Ljubljana, Aškerčeva 7, 1000 Ljubljana, Slovenia; Matej.Sova@ffa.uni-lj.si; 5 Department of Pharmaceutical Technology, Faculty of Pharmacy, University of Ljubljana, Aškerčeva 7, 1000 Ljubljana, Slovenia; Julijana.Kristl@ffa.uni-lj.si; 6 Department of Physiology and Pharmacology “Vittorio Erspamer”, Sapienza University, P.le Aldo Moro 5, 00185 Rome, Italy; luciano.saso@uniroma1.it

**Keywords:** NRF2 modulators, anticancer agents, nanoparticles, drug delivery, drug targeting

## Abstract

The disadvantages of conventional anticancer drugs, such as their low bioavailability, poor targeting efficacy, and serious side effects, have led to the discovery of new therapeutic agents and potential drug delivery systems. In particular, the introduction of nano-sized drug delivery systems (NDDSs) has opened new horizons for effective cancer treatment. These are considered potential systems that provide deep tissue penetration and specific drug targeting. On the other hand, nuclear factor erythroid 2-related factor 2 (NRF2)-based anticancer treatment approaches have attracted tremendous attention and produced encouraging results. However, the lack of effective formulation strategies is one of the factors that hinder the clinical application of NRF2 modulators. In this review, we initially focus on the critical role of NRF2 in cancer cells and NRF2-based anticancer treatment. Subsequently, we review the preparation and characterization of NDDSs encapsulating NRF2 modulators and discuss their potential for cancer therapy.

## 1. Introduction

Cancer has long been a focus of scientific research due to its high incidence and mortality rate. Oxidative stress has been associated with cancer formation, increased tumor aggressiveness, and cellular resistance to therapy. Nuclear factor erythroid 2-related factor 2 (NRF2) is a transcription factor that primarily regulates the endogenous cellular antioxidant defense system and has been shown to play an effective role in cancer treatment. The tumor-suppressive or pro-oncogenic function via NRF2 modulation (inhibition or activation) has emerged as an approach to cancer prevention and therapy in the form of personalized medicine. 

Many research studies have shown that the suppression of NRF2-related antioxidant mechanisms is a viable approach for inducing pro-oxidizing conditions in the tumor microenvironment and can trigger reactive oxygen species (ROS)-induced cell death in a variety of tumors and organs. However, numerous challenges limit the clinical usage of NRF2 modulators, such as poor solubility and membrane permeability, low bioavailability, poor in vitro and in vivo stability, a short half-life, rapid metabolism, and rapid clearance [1,2]. 

Developments in pharmaceutical nanotechnology promise the circumvention of these drawbacks and to improve the delivery of NRF2 modulators via nano-sized drug delivery systems (NDDSs). Nanoparticles, polymeric micelles, and liposomes are among the promising NDDSs designed to improve the solubility and facilitate the targeting of NRF2 modulators and, thus, lead to reduced toxicity in healthy tissues. The design of ROS-sensitive NDDSs improves the efficacy in targeting the tumor microenvironment with abnormal ROS levels. In addition, the combined administration of NRF2 modulators and conventional antineoplastic agents via NDDSs can provide dual drug release and lead to innovative paradigms in the treatment of drug-resistant tumors. 

This review provides an overview of these NDDSs including their benefits, structure, preparation, characteristics, and applications. In addition, the performance and future development of NDDSs are discussed.

## 2. Critical Role of NRF2 in Cancer Cells

NRF2 is one of the key players in the maintenance of cellular homeostasis through controlling the redox balance, metabolism, proliferation, and protein folding [3]. In the last 10 years, there have been several publications that indicated cancer as being closely connected to the NRF2/KEAP1 signaling pathway [4,5,6,7,8,9]. However, when targeting the NRF2/KEAP1 signaling pathway for the prevention and potential treatment of cancer, the dual roles of NRF2 in cancer development have to be considered [7,10,11].

Under normal physiological conditions, the protective role of NRF2 acting as a tumor suppressor via maintaining cellular redox homeostasis (eliminating ROS and carcinogens) and regulating cell growth is acknowledged, whereas, in many established cancers, upregulation of NRF2 was detected, thereby assisting cancer cells to withstand excessive oxidative stress and diminishing the effects of chemotherapeutic agents and radiotherapy [5,7,11]. Indeed, NRF2 was proven to be responsible for the radio- and chemoresistance of cancer cells (mainly by stimulating drug metabolism or drug efflux) in addition to inflammation-induced carcinogenesis [11,12]. 

NRF2 also stimulates cancer cell growth and proliferation, promotes sustained angiogenesis, and suppresses cancer cell apoptosis. NRF2 is recognized as a pleiotropic transcription factor that is also critically involved in metabolic reprogramming in cancer cells [9]. Numerous other beneficial or detrimental effects of NRF2 acting via diverse signaling pathways have also been reported and extensively reviewed [9,12,13,14]. All facts mentioned confirm that NRF2 possesses a critical role in cancer and, thus, represents a very promising future target for anticancer treatment.

## 3. NRF2-Based Anticancer Treatment

The potential anticancer activity as a result of NRF2/KEAP1 pathway targeting could be accomplished either by positive modulation of KEAP1, a negative regulator of NRF2, by inhibition/activation of NRF2, or by affecting/disrupting the NRF2/KEAP1 protein–protein interactions [7,15]. The design of KEAP1/NRF2 protein–protein interaction inhibitors might be advantageous due to the potential improvement in target selectivity [16]. These inhibitors can be divided into peptidic and non-peptidic/small-molecule.

Peptides are less appropriate as potential anticancer agents due to their undesirable pharmacokinetic properties; however, some efforts in enhancing their affinity, selectivity, and stability have been made [17]. The crystal structures of the KEAP1 Kelch domain and NRF2 peptides enable different design strategies and have promoted the research and development of KEAP1/NRF2 inhibitors [7,17,18,19,20]. The majority of NRF2 research is, thus, currently focused on the design of small-molecule inhibitors, which possess greater potential in terms of stability, safety, and pharmacokinetic properties.

**Figure 1:**
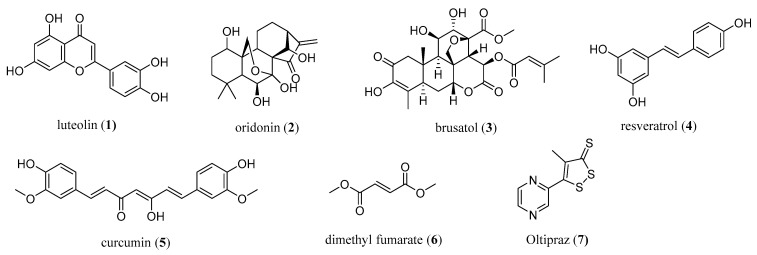
The most common natural (**1**–**5**) and synthetic (**6**,**7**) NRF2 modulators.

The modulation of the activity of NRF2 has emerged as a novel modern strategy for cancer chemoprevention and therapy. Currently, numerous NRF2 modulators have been discovered and are either natural compounds (e.g., luteolin (**1**), oridonin (**2**), brusatol (**3**), resveratrol (**4**), curcumin (**5**)), peptides, or small-molecule semi-synthetic and synthetic compounds (Figure 1) [7,15]. Natural compounds targeting NRF2 are of interest for cancer intervention, especially in terms of their chemopreventive effects as well as in preventing the chemoresistance of cancer cells, thereby enhancing the cytotoxic effects of known chemotherapeutic agents [7].

However, the majority of natural NRF2 modulators suffer either from undesirable off-target effects, potential toxicity, or poor physicochemical properties (e.g., low solubility, poor in vitro and in vivo stability) affecting the membrane permeability and leading to a rapid metabolism, a short half-life, and clearance. Several analogs of natural compounds have been prepared in order to ameliorate the physicochemical properties and/or lower toxicity, and these were systematically reviewed three years ago [7].

To date, only one NRF2 activator, dimethyl fumarate (**6**) (under the trade name Fumaderm or Tecfidera), has been used clinically for the treatment of psoriasis and remitting relapsing multiple sclerosis [7,16] (Figure 1). Many recent and older studies also showed the antiproliferative and proapoptotic properties of dimethyl fumarate [7,21], which also exhibited antitumor activity in cellular and mice models [22]. A dose-dependent anticancer mechanism was proposed [22].

Based on the recent crystal structures and binding studies, it was suggested that dimethyl fumarate binds at multiple KEAP1 binding sites, thus activating NRF2 through the covalent as well as the noncovalent mode of action [23]. The therapeutic potential of dimethyl fumarate in cancer still requires investigation, and more data from in vivo studies in humans are required. Another NRF2 modulator from clinical trials is oltipraz (**7**), which is commonly related to its potential cancer-preventive interventions targeted to the colon and liver [24].

Although many compounds that could modulate the NRF2/KEAP1 pathway are known, there is still a high demand for additional in vitro and in vivo studies to clarify the mechanism of NRF2 modulation in cancer cells and predict the potential effects (positive or negative) of interfering with the NRF2/ KEAP1 pathway [7]. Recent studies have also focused on nano-sized drug delivery systems (NDDSs), which will be discussed in the following sections.

In addition to small-molecule NRF2 modulators, there is also a promising therapeutic approach via siRNA-mediated targeting of the NRF2 pathway. Small interfering RNAs (siRNAs) are RNA-based therapeutic oligonucleotides that can inactivate several target RNAs in a sequence-specific manner, thus showing the potential to be used in the silencing of any cancer-related genes in addition to the targeting of different molecular pathways [7,15,25,26].

For example, bioengineered NRF2-siRNA molecules were capable of decreasing the NRF2 mRNA and protein levels in human osteosarcoma cells, leading to the reduced expression of NRF2-regulated oxidative enzymes and higher intracellular ROS levels [27]. Researchers found that NRF2 knockdown by siRNA transfection was able to enhance the oxidative damage and apoptosis induced by cisplatin, suggesting a new potential approach for human laryngeal squamous carcinoma treatment [28]. Thus, siRNA-based therapeutic agents show great potential; however, the stability of modified versions of siRNAs and the specificity and efficiency of siRNA delivery into cancer cells, along with unwanted immune responses, need to be improved in the future [7,15,25].

## 4. NDDSs in the Delivery of NRF2 Modulators for Cancer Therapy

Numerous formulation development strategies have been developed to improve the overall bioavailability of antineoplastic agents by providing the delivery of drugs to target sites at the required concentrations within a certain time. In particular, nanotechnology-based drug delivery methods provide a promising prospect for the prevention, diagnosis, and treatment of cancer patients [29]. Drugs with various solubility profiles can be either encapsulated or conjugated into NDDSs. These systems are well tolerated in vivo due to their biocompatible and biodegradable nature.

**Figure 2:**
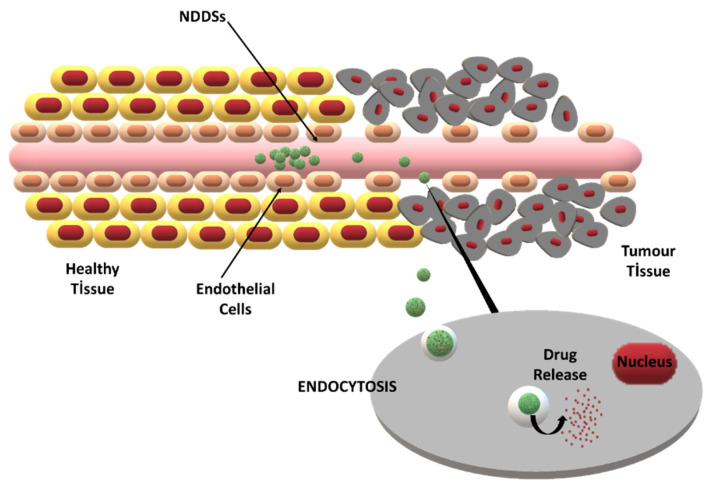
The enhanced permeability and retention (EPR) effect and its role in passive drug targeting to tumor cells via NDDSs.

The major challenge in tumor-specific drug delivery is to provide drug transport through biological barriers [30]. By modifying the formulation and manufacturing process parameters, the characteristics of NDDSs, such as the particle size, drug loading, and surface charge, can be tailored for specific applications. Thus, improved barrier penetration and drug targeting can be obtained [31]. The two main targeting strategies for NDDSs are passive targeting via the enhanced permeability and retention (EPR) effect and active targeting via site-specific ligands (Figure 2).

The controllable drug release behavior of NDDSs provides good therapeutic activity. In recent decades, theranostic NDDSs that combine imaging as well as therapeutic functions were developed to augment success in cancer therapy [32]. NRF2 modulators have demonstrated tumor-suppressive and pro-oncogenic functions. However, their inability to differentiate tumorous tissue leads to poor biological activity and side effects in healthy tissue. Different NDDSs reported for the efficient delivery of NRF2 modulators will be described below and their advantages will be reviewed. The limiting challenges for the clinical usage of various NRF2 modulators and the current approaches to circumvent these difficulties via NDDSs are also discussed.

### 4.1. Liposomes

**Figure 3:**
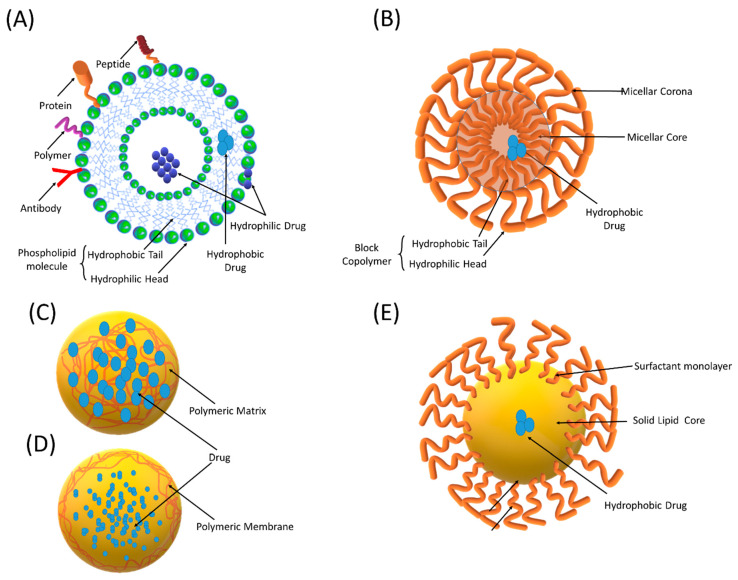
Schematic representation of different nano-sized drug delivery systems (NDDSs): (**A**) liposomes, (**B**) polymeric micelles, (**C**) nanospheres, (**D**) nanocapsules, and (**E**) solid lipid nanoparticles.

Liposomes are microscopic spherical vesicles composed of concentric lipid layers containing one or more aqueous compartments (Figure 3). They were first identified by Bengham et al. in the mid-1960s [33]. The characteristic lipid bilayers of liposomes are composed of natural phospholipids and cholesterol that resembles a cellular plasma membrane. They can encapsulate a variety of drugs, including small-molecule entities, genetic materials, and enzymes within their internal aqueous core or in the lipid bilayer. Their ability to protect and target the encapsulated drug renders liposomes attractive for biomedical applications.

The size of liposomes ranges from 30 nm to a few microns and plays a pivotal role in determining the in vivo fate of a system. The developments in liposome technology have led the clinical usage of liposome-based preparations, such as Doxil^®^, DaunoXome^®^, and Depocyt^®^, and more are in clinical trials [34]. By modifying the lipid composition, size, and surface charge of liposomes, circulating and tissue-specific systems can be prepared. Although industrial scalability, reproducibility, and stability are challenging limitations of liposomal drug delivery, they are still accepted as innovative systems. The intratumor accumulation of liposomes is provided by the EPR effect.

Accordingly, an abnormal tumor vasculature with leaky capillary basement membranes enables the penetration of liposomes through blood vessels and leads passive drug targeting. Liposomes can be actively targeted to tumor cells by conjugating targeting moieties (small-molecule ligands, peptides, or monoclonal antibodies). The cellular liposome uptake by endocytosis provides improved drug efficiency. Intracellular and organelle targeting via liposomes expands the therapeutic window. Recent developments in liposome technology gave rise to stimuli (pH, temperature, redox, and enzyme)-sensitive liposomal systems that provide better anticancer efficacy [35].

Luteolin, a plant-derived flavonoid, was shown to sensitize specific carcinoma cells to chemotherapeutic drugs (oxaliplatin, bleomycin, and doxorubicin) by inhibiting NRF2 [7,36,37] (Figure 1). Although the antitumoral potential of luteolin is discussed in the literature, it is not clinically available for several reasons. Low aqueous solubility and excessive excretion are the primary drawbacks [38]. Luteolin-loaded D-alpha tocopheryl polyethylene glycol 1000 succinate (TPGS)-coated liposomes with a particle size of less than 200 nm demonstrated improved cytotoxicity and cellular uptake in lung carcinoma cells (the A549-human adenocarcinoma alveolar basal epithelial cell line).

A significant reduction in tumor size and systemic side effects was observed upon liposomal luteolin administration to A549 tumor-bearing nude mice. A significant increase in the antitumoral effect of luteolin was attributed to a prolonged drug residence time in the blood and tumor-targeted drug delivery via liposomes [38]. Lesitin-based luteolin-encapsulated liposomes inhibited the activity of tumor growth more effectively than free luteolin in both the CT26 colorectal carcinoma cell line and a mouse tumor model [39].

Ferroptosis is a newly recognized type of cell death that differs from conventional necrosis, apoptosis, or autophagic cell death. This type of cell death is characterized by low glutathione and high lipid ROS levels. Ferroptosis has been considered as an antitumoral treatment; however, the NRF2 overexpression in tumor cells hinders cell death. Erastin is a ferroptosis inducer that selectively kills non-small cell lung cancer cells (NSCLC), but its usage is hindered due to poor solubility. Folat-modified liposomes co-encapsulating erastin and the metallothionein 1D pseudogene (an NRF2 inhibitor) were shown to have significant antitumor effects on NSCLC xenografts compared to erastin. Gene–drug co-delivery via liposomes hampered the proliferation, migration, and invasion of ferroptosis-insensitive NCSLC in cell culture studies. [40].

Recent data revealed the antitumoral effects of oridonin, a diterpenoid isolated from Rabdosia rubescens [15,41] (Figure 1). Poor water solubility, ease of oxidation, a short biological half-life, and a low therapeutic index are major obstacles in the preparation of effective oridonin formulations [42]. Liposomes are considered as novel drug carrier candidates to overcome these problems [42,43,44].

Oridonin was formulated in long-circulating stealth liposomes obtained by the inclusion of polyethylene glycol (PEG) on the liposomal surface. PEGylation increases the hydrophilicity of the liposome surface, provides steric stabilization, and, thus, escapes from RES clearance. An improved tumor inhibition rate and reduced toxicity were observed with this method compared to oridonin solution. The receptor-mediated cellular uptake of nanoliposomes into HepG-2 tumor cells was provided by folate conjugation. This strategy provided higher tumor inhibition (85.6%) in BALB/c nude mice compared to unconjugated liposomes (66.8%) and free oridonin [45].

In a recent study, sonodynamic therapy was studied in combination with oridonin chemotherapy as a potential liver cancer treatment. Oridonin liposomes containing microbubbles were covalently adhered to folic acid-conjugated protohemin-loaded multiwalled carbon nanotubes. Internalization of the construct was provided via folate receptor endocytosis, and drug release was triggered by sonication, leading to a synergetic anticancer activity [42].

Plumbagin, a natural naphtoquinone, is known to induce apoptosis in various cancer cells, such as pancreas, lung, breast, and prostate cancer cells. This effect is induced by reduction in the NRF2 nuclear translocation that leads to excessive ROS production. Although plumbagin has anticancer potential, its toxicity and short biological half-life in healthy tissues are important problems. Serious side effects in routine applications include bleeding, diarrhea, skin rashes, and hepatic toxicity. Several formulation strategies were developed to improve the in vivo efficiency of plumbagin using liposomal drug delivery systems (Table 1).

Two NRF2 activators, curcumin and resveratrol, have also been formulated in liposomal form to enhance their in vivo efficacy (Figure 1). Arginine, glycine, aspartic acid peptide (RGD)-modified paclitaxel, and curcumin co-loaded liposomes were effective in the inhibition of angiogenesis and metastasis of A549 lung cancer cells [51]. Didecyldimethylammonium bromide-containing liposomal curcumin improved the anticancer efficiency and apoptosis effect on HeLa and SiHa cells, indicating the potential of this system for the treatment of cervical cancers [52].

The mitochondrial targeting of resveratrol was achieved via a dequalinium polyethylene glycol-distearoylphosphatidylethanolamine (DQA-PEG2000-DSPE) liposomal formulation, and the apoptosis of both non-resistant A549 cells and resistant A549/cDDP cells was induced [53]. Many other liposomal formulations of curcumin and resveratrol are also reported in the literature, and their combinations were studied as well [54,55,56,57].

### 4.2. Polymeric Nanoparticles

Polymeric nanoparticles (PNPs) are defined as colloidal particles with sizes ranging from 1 to 1000 nm. Biocompatible and biodegradable polymers with natural or synthetic origin are used to form the PNP structure. PNPs are alternative systems to liposomes with very similar shape and size properties; however, they provide additional advantages, such as better in vivo/in vitro stability, high cargo capacity, and targeting. Biodegradable polymers, such as polyesters (Poly(lactide-co-glycolide)-PLGA, poly-ε-caprolactone-PCL), polyamides (gelatin, albumin, etc.), polyanhydrides, polyurethanes, and polyphosphazenes, have been used to prepare PNPs [58].

Among these polymers, the FDA-approved PLGA is promising due to its biodegradability and biocompatibility properties [59,60]. Lab-scale PNP production methods include coacervation, emulsion solvent evaporation/extraction, salting out, emulsification, solvent diffusion, and emulsion polymerization. Depending on the preparation method, PNPs with optimum characteristics can be obtained using suitable stabilizing agents (poly(vinyl alcohol)-PVA, pluronics, etc.) and solvent systems (acetone, dichloromethane, chloroform, etc.).

Techniques such as spray drying and supercritical fluid spraying can be employed for the large-scale production of PNPs [61]. In the literature, the term PNPs is used for nanocapsules and nanospheres that differ from each other by their morphological structures [62]. Nanocapsules are reservoir systems, which encapsulate the dissolved drug in a liquid core that is surrounded by a polymeric membrane (Figure 3). On the other hand, nanospheres are polymeric matrix systems that entrap a drug inside their network or on the surface.

These are known as first-generation PNPs that passively target tumor cells. The advances in pharmaceutical R&D studies have created the new generation of PNPs for carrier-mediated drug targeting to cancer cells [63]. In this respect, second-generation nanoparticles were developed by coating PNPs with polymers that specifically bind to the overexpressed receptors on cancer cells. Third-generation PNPs, on the other hand, contain cell-specific ligands for specific targeting. This new generation of PNPs provides active drug targeting to the tumor site.

The particle size, zeta potential, entrapment efficiency, and drug release profile of nanocarriers are the most important critical material attributes that affect the in vivo effectiveness. These characteristic properties of PNPs can be tailored by modifying the formulation (polymer type, solubility, molecular weight, excipient type, drug ratio, etc.) and manufacturing process (homogenization speed, temperature, etc.) parameters [64]. Based on the formulation design, the release rate of anticancer drugs from PNPs can be controlled by different mechanisms, including drug diffusion, erosion, hydrolysis, and the enzymatic degradation of the polymer matrix.

As a natural quassinoid, brusatol is known to stimulate the polyubiquitination of NRF2 and inhibit NRF2 signaling [65] (Figure 1). Studies suggest its usage as an antineoplastic drug and chemotherapeutic adjuvant [66,67]. However, brusatol causes various cardiovascular side effects and may lead to serious neurologic and organ damage by reversing the therapeutic effects of other drugs. The emerging need for new therapy regimens for cancer patients led researchers to reduce the side effects of brusatol via NDDSs. Chen et al. loaded brusatol in PLGA-based nanoparticles conjugated with glycosaminoglycan-placental chondroitin sulfate A-binding peptide (plCSA-BP) [68]. The nanoparticles efficiently promoted cancer cell apoptosis by regulating the B cell CLL/lymphoma 2 (BCL2), BCL2-associated X protein BAX, cleaved caspase-3, matrix metalloproteinase (MMP)-2, and MMP-9 pathways. The efficiency of nanoparticles was higher compared to pure brusatol. In another study, brusatol-loaded mPEG-PLA nanoparticles were shown to provide sustained brusatol release and lead to improved cytotoxicity on prostate cancer cell lines (PC-3 and LNCaP) compared to the pure free drug. This result was attributed to the internalization of the nanoparticles in PC-3 cells reaching deep inside the cells [69].

Another negative modulator of NRF2 signaling is trigonelline. This alkaloid compound is a metabolite of nicotinic acid and was shown to have anti-invasive activity against cancer cells. It is an ideal drug candidate to treat neurodegenerative diseases and cancer. One of the main obstacles with trigonelline administration to treat brain tumors is the presence of the blood–brain barrier (BBB), which prevents influx of the drug. To overcome this problem, trigonelline-loaded chitosan nanoparticles (Trigo-WSCS NPs) were prepared by ion complex formation.

Microscopic and flow cytometric analysis confirmed apoptotic cell death for C6 glioma cells via Trigo-WSCS NPs [70]. Trigo-WSCS NPs provided enhanced biocompatibility and neurite growth of rat adrenal pheochromocytoma (PC12) cells. Another research group demonstrated the inhibitory effect of trigonelline-incorporated chitosan nanoparticles on tumor cell (CT26 colon carcinoma) invasion [71]. The antioxidant properties of chitosan are well known and the improved inhibition of cell invasion was explained by a possible synergistic effect of trigonelline and chitosan.

As a natural flavone, chrysin is recognized as an antitumor agent due its NRF2 inhibition mechanism mediated through the PI3K/Akt pathway [37,72]. Chrysin suppress the proliferation, migration, and invasion of various cancer cells; however, its bioavailability and biomedical applications are limited due to its poor aqueous solubility, low permeability, and rapid metabolism. Therefore, researchers performed many nanocarrier-based formulation studies for chrysin [73,74,75].

A pH-responsive poly ((lactide-co-glycolic acid)-block-methacrylic acid) (PLGA-co-PMAA)-based nanoparticle formulation was designed to co-deliver doxorubicin and chrysin for cancer therapy. The nanoparticle formulation allowed the pH-responsive dual release of both drugs and significantly decreased the viability of human lung epithelial cancer cell lines (A549). The observed cytotoxicity effect was significantly improved compared to the free drugs or single drug-loaded nanoparticles [76].

The inhibitory effects of the apigenin flavonoid on cancer cells were demonstrated; however, its bioavailability is poor due to its low aqueous solubility and stability. Apigenin lacks a specific targeting effect on tumor cells, and light exposure, the presence of oxygen, and harsh conditions, such as high temperature, cause stability problems. Therefore, researchers worked on a wide range of drug delivery systems to improve both the stability and bioavailability of apigenin [77,78,79].

Apigenin-encapsulated mesoporous SiO_2_-coated Fe_2_O_3_/Fe_3_O_4_ heterogeneous magnetic nanoparticles were surface modified with polysaccharide hyaluronic acid. The magnetic nanocomposite system provided magnetic targeting and active hyaluronic acid targeting. This system led to the enhanced therapeutic activity of apigenin via the promotion of apoptosis, lipid peroxidation, and ferroptosis [79].

Another NRF2 inhibitor, wogonin, was isolated from the roots of Scutellaria baicalensis Georgi. Wogonin was shown to be cytotoxic against cancer cells, such as nasopharyngeal carcinoma cells (NPC) [80] and hepatocellular carcinoma (HCC) [81], and its efficacy in inhibiting tumor invasion was also reported [82]. One of the main formulation problems of wogonin is its poor aqueous solubility [83]. Wogonin was conjugated to magnetic (magnetite Fe_3_O_4_) nanoparticles to improve its solubility and therapeutic efficiency. This system was found to induce the apoptosis of Raji cells by blocking the G0/G1 phase of the cell cycle and was suggested as a promising therapy strategy in lymphoma [84].

The treatment potential of photodynamic therapy in cancer is hampered due to the activation of intracellular antioxidant responses. In order to improve the efficacy of PDT against oral tongue squamous cell carcinoma, Shi et al. prepared a poly(β-amino ester)/poly lactic-co-glycolic acid-based nanoparticle system encapsulating photosensitizer indocyanine green (ICG) and NRF2-siRNA [85]. This nanoparticle system was further coated with a homologous cancer cell membrane to provide biomimetic properties to the nanoparticles. The results of the study revealed inhibited proliferation and induced apoptosis of tumor cells via nanoparticle targeting and also confirmed the synergist effect of NRF2-siRNA for amplifying PDT by down-regulating NRF2 expressions.

### 4.3. Solid Lipid Nanoparticles

Solid lipid nanoparticles (SLNs) can be defined as colloidal dispersions composed of lipids that are solid at room and body temperature (Figure 3). SLNs were developed as alternative carriers to liposomes and polymeric nanoparticles in order to overcome the disadvantages, such as polymer degradation, cytotoxicity, large-scale manufacturing problems, drug leakage, and instability [86,87]. The solid lipid core of SLNs forms a matrix that can encapsulate both hydrophobic and lipophilic drugs. The outer shell of SLNs is made up of surfactants (phosphatidylcholine, lecithin, and pluronics) that stabilize the system.

Stearic acid, glyceryl behenate (compritol), cholesterol, caprylic/capric triglyceride, glyceryl stearate, and solid paraffin are among the commonly used SLN-forming lipids [88]. They also protect the incorporated drug against chemical, photochemical, or oxidative degradation and prevent its leakage while additionally offering the possibility of sustained release, as the drug’s mobility in the solid lipid phase is considerably lower than that in the oily phase [89,90]. SLN preparation methods include high-pressure homogenization, hot/cold homogenization, double emulsion, and solvent displacement and emulsification–diffusion techniques. Drug loading and distribution into SLNs depends on the lipophilicity and structure of the molecules [91]. They are widely studied for cancer therapy.

SLN formulations of oridonin were prepared using different lipid compositions to improve the in vitro release behavior, in vivo pharmacokinetics, and tissue distribution profile [92,93,94]. In cell culture studies, oridonin-containing SLNs inhibited the proliferation of cancer cells (MCF-7, HepG 2, and A549) more effectively compared to the free drug and, promisingly, did not exhibit any significant toxicity enhancement on human mammary epithelial cells [93]. Extended cellular uptake degrees and times improved the antitumoral effects of SLNs. In addition, SLNs induced higher apoptotic rates compared with the free drug by inducing significantly more cell cycle arrest at S and decreasing the cell cycle arrest at the G1/G0 phase.

Sun and coworkers investigated the effect of astraxanthin in SLNs on the incidence of breast cancer. Numerous investigated biochemical parameters have shown that astraxanthin in SLNs reduces the incidence of tumors and has a chemoprotective effect on breast cancer via the NRF2/KEAP1 mechanism [95].

A study focused on the health-protective effects of resveratrol, a naturally occurring polyphenol that shows pleiotropic health-beneficial effects, including antioxidant, anti-inflammatory, anti-aging, cardioprotective, and anticarcinogenic effects. The researchers found that these therapeutic effects of resveratrol in SLNs were associated with the modulation of the NRF2 signaling pathway [96].

### 4.4. Polymeric Micelles

In recent years, polymeric micelles have received great attention as promising drug delivery systems for therapeutic compounds. They are ideal carriers especially for molecules with low solubility and high potency and toxicity [97,98]. Polymeric micelles are self-assembling colloidal nanocarriers that consist of a hydrophobic core and a hydrophilic corona. One or more drugs can be solubilized in the micellar core; thus, a synergistic anticancer activity can be obtained [99]. Targeting of polymeric micelles to the tumor site can be achieved by attaching targeting moieties to the micellar corona [100].

Micelle-forming copolymers consist of hydrophobic polymers (poly(amino acids), polyesters, and phosphatidyl ethanolamines) and hydrophilic polymers (poly(ethylene glycol), poly(oxazolines), chitosan, and dextran) [101]. Drug-conjugated polymers can also form micellar structures and modify the drug release profile [102]. Micelles are very attractive drug delivery systems for cancer therapy due to their small particle size (<100 nm), which enables tumor-selective targeting via the EPR effect.

In addition, they are sensitive to tumor microenvironmental stimuli, such as the pH, hypoxia, and enzymes [101,103]. As a response to low tumoral pH, the pH-sensitive polymers (e.g., poly-β-aminoester and polyhistidines) or pH-dependent degradable linkers (e.g., hydrazones) of micelle-forming polymers may lead micellar disassembly and trigger the release of the encapsulated cargo. This phenomenon is used for various anticancer agents, including gambogic acid [104], paclitaxel [105], doxorubicin [106,107], and gefitinib [108].

The unorganized growth of the vasculature with rapid and uncontrolled proliferation of tumors limits the oxygen supply in tumor cells. As a result, the upregulation of hypoxia markers leads the formation of hypoxic regions around solid tumors [109]. The addition of hypoxia-sensitive groups (nitroimidazole) to the structure of micelle-forming copolymers may provide the controlled release of hydrophobic drugs under hypoxic conditions of tumor cells [109].

A micelle-forming amphiphile prepared by grafting Pluronic^®^F68 with linoleic acid (LA) moieties via disulfide bonds (F68-SS-LA) was used to encapsulate brusatol. The tumor microenvironment-responsive brusatol-loaded micellar carrier was found to exhibit rapid particle dissociation and drug release in response to a redox environment. In addition to its unique drug release ability, the good stability and small particle size of the micellar system improved the cellular internalization and cytotoxicity in both Bel-7402 and MCF-7 cells compared with free brusatol [110].

To overcome oxaliplatin resistance in colon cancer cells, Tazehkand et al. studied the potential of trigonelline and trigonelline-loaded micelles on the inhibition of NRF2. Micellar formulations of PCL-PEG-PCL (3Block) and PLA-PCL-PEG-PCLPLA (5Block) copolymers entrapped trigonelline with a high encapsulation efficiency (95% and 92%, respectively).

Micellar trigonelline was found to be more effective in the functional inhibition of NRF2, leading to improved impacts on drug resistance in SW480 colon cancer cells. AnnexinV/PI flow cytometry analysis and apoptosis-related gene expression analysis (Bax) revealed that apoptosis was significantly induced by trigonelline-loaded 5Block micelles compared to free trigonelline and 3Block micelles [111]. The success of the 5Block micelles was attributed to the lower crystallinity of PLA in their structure, which provides higher sensitivity to the temperature and higher degradation.

Apigenin-encapsulated mixed polymeric micelles composed of Pluronic P123 and Solutol HS15 exhibited a sustained release profile. The obtained micellar system was found to be stable against dilution due to its low critical micelle concentration. In vitro cytotoxicity studies showed an enhanced inhibiting effect of the micellar formulations on the growth of HepG2 and MCF-7 tumor cells [78].

A micelle-based nano-platform was prepared for the combined delivery of rapamycin and wogonin to improve the cytotoxic effects on MCF-7 breast cancer cells. This core–shell structure consisted of a zein–lactoferrin copolymer that was cross-linked with glutaraldehyde. The micellar system provided sequential drug release for two different drugs. Both the physical and serum stabilities of the combined micellar system were shown. The cellular uptake and in vitro cytotoxicity tests on MCF-7 breast cancer cells revealed a superior antitumor effect of the cross-linked micelles compared to free drugs [112]. This was attributed to the superior micellar stability, and a better targeting ability that led to the enhanced tumoral accumulation of micelles.

### 4.5. Nanoemulsions

Among other lipid-based NDDSs, great progress has been made in nanoemulsified systems. They tend to be less toxic than other NDDSs and present added advantages, such as easy preparation, biodegradability, biocompatibility, and thermodynamic stability, to provide adequate protection of the NRF2 modulators from degradation and controllable release [113,114].

Nanoemulsions have been used as a successful formulation for clinical and therapeutic applications, and they can be administered via a diversity of routes including via intranasal administration to target the central nervous system (CNS) and intravenous route to deliver NRF2 modulators directly to the systemic circulation. Clinical trials have been reported using nanoemulsions (clinicalTrial. Gov ID NCT02367547, NCT03865992, NCT01975363) [115], although the number of nanoemulsions in clinical studies and accepted for therapeutic use is still limited.

Nanoemulsions are artificially generated NDDSs composed of water, oil, and emulsifiers in proper proportions, normally 20–100 nm in diameter, that have been proven to be sufficiently small to cross blood vessels and sufficiently large to promote rapid renal clearance [115]. Miniaturization of the droplets enhances the EPR effect, improving the pharmacokinetics and pharmacodynamics of the nanoencapsulated NRF2 modulators.

Typically, nanoemulsions are composed of natural ingredients, such as vegetable oils (olive, soybean, corn, etc.) and/or fish oils, containing examples of long-chain triglycerides, and phospholipids (soy lecithin), proteins (caseinate), or Tweens (Tween 80), examples of emulsifiers. They can mainly be formed in two ways: (1) the oil dispersed in the continuous aqueous phase (O/W) or (2) the aqueous phase dispersed in the continuous oil phase (W/O).

Conceptually, it is possible to distinguish three regions in a nanoemulsion (the lipid, aqueous, and interfacial regions) in which NRF2 modulators could be loaded for delivery to tumor sites according to their polarity and surface activity [116]. The different chemical properties of different regions make NDDSs ideal for encapsulating different NRF2 modulators of different solubilities, for instance, hydrophilic compounds can be loaded within the aqueous region, while hydrophobic NRF2 modulators can be located within the hydrophobic oil core, and modulators of intermediate hydrophobicity can be located in the interfacial region of the nanoemulsion [116].

**Figure 4:**
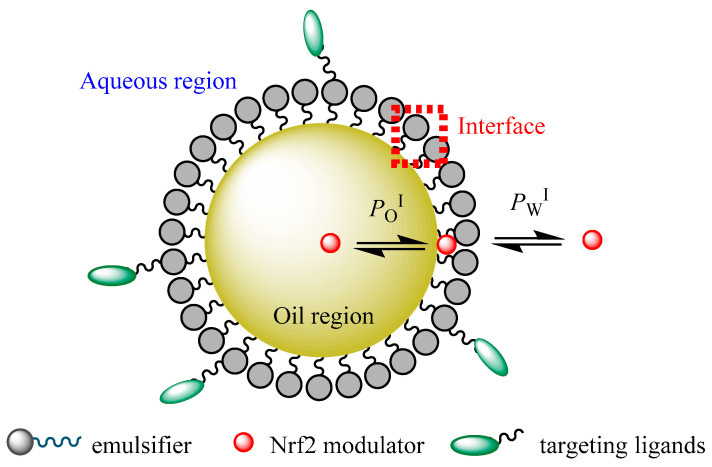
A schematic representation of a functionalized O/W nanoemulsion and distribution of a potential NRF2 modulator between their different regions (aqueous, interfacial, and oil regions). *P*_W_^I^ and *P*_O_^I^ stand for the partition constants between aqueous–interfacial and oil–interfacial regions, respectively.

A schematic representation of the partitioning of an NRF2 modulator between the different regions of a functionalized nanoemulsion is shown in Figure 4. Thus, nanoemulsions can carry modulators of different solubilities to a target site, thus improving their bioavailability. Understanding the distribution of potential NRF2 modulators between different microenvironments of nanoemulsions and the factors that control their distribution (formulation and environmental properties) will contribute to their clinical success and the clinical setting of cancer therapy [116].

An appropriate formulation of a nanoemulsion is considered a valuable strategy for enhancing the protection of NRF2 modulators from degradation and affording improved absorption and bioavailability. For example, resveratrol can be easily degraded when it is located mainly in the interfacial region of nanoemulsions, and the composition of the nanoemulsified system must be optimized to improve its incorporation into the oil region [117].

These NDDSs not only overcome modulator solubility issues but also allow the tumor-specific targeting of NRF2 modulators and may be proposed to solve multidrug resistance problems. Additionally, nanoemulsions can be modified by anchoring targeting ligands to the interface, providing functional and multifunctional nanoemulsions that may overcome the issues of delivering modulators to multidrug-resistant cancer cells, recognizing certain compounds on the cancer tissue [118], Figure 4.

They can be designed to control their function and to select a specified target. Attempts have also been made to deliver NRF2 modulators via nanoemulsion with a beneficial effect with in vitro and/or in vivo oral chemotherapy [72]. Phenolic compounds, such as curcumin and resveratrol, are potential NRF2 modulators in cancer prevention and therapy [72], and their chemopreventive properties can be enhanced by encapsulation in nanoemulsions. Curcumin, (1E,6E)-1,7-bis(4-hydroxy-3-methoxyphenyl)hepta-1,6-diene-3,5-dione, is a natural hydrophobic polyphenol that presents in the dried rhizomes of the medicinal plant Curcuma longa (turmeric), and its biological and pharmacological activities have been widely reported [119,120].

Several studies demonstrated the ability of curcumin to reactivate the expression of antioxidant enzymes, such as glutathione S-transferase, aldose reductase, and HO-1, through KEAP1/NRF2 signaling [119]. Curcumin could be a possible NRF2 activator to regulate the cell signaling pathway in stress environments and be beneficial in treating several oxidative stress-related disorders, such as cancer (including breast, gastrointestinal, and prostate), neurodegenerative diseases (NDs), autoimmune diseases, and cardiovascular diseases (CVDs), among others [119,120].

However, the clinical applications of curcumin remain unsatisfactory due to its poor aqueous solubility (log *P*_W_^OCT^ = 3.29 [121]), chemical stability at physiological pH (e.g., it can be hydrolyzed within the intestine, pH 6.8), poor absorption into the small intestine epithelial cells due to its lipophilic nature, fast metabolism in the liver and small intestine prior to entering the systemic circulation, and rapid elimination from the body. These drawbacks may be overcome by encapsulating curcumin in an oil-in-water (O/W) nanoemulsion [120].

Although several encapsulation alternatives have been considered for curcumin [122], nanoemulsion was noted as the most convenient encapsulation choice, preserving its chemical structure and enhancing its aqueous solubility and bioavailability. Curcumin is not aqueous soluble and, thus, is only distributed between the oil and interfacial regions of nanoemulsions, being protected from active compounds located in the aqueous region that may promote its degradation.

Several studies reported that curcumin encapsulated in nanoemulsions can significantly increase its bioavailability compared to free curcumin [120]. Related to the anticancer activity of curcumin nanoemulsions, in vitro and in vivo experiments reported that nanoemulsions could boost the anticancer activity [120]. For example, Guerrero et al. [123] reported on curcumin encapsulated in O/W nanoemulsions to treat the growth of metastatic development of cancer.

They evaluated its efficacy and toxicity in syngeneic B16F10 cells in C57BL/6 mice. The results showed that curcumin in nanoemulsions decreased the in vitro cell proliferation in tumor cells rather than in benign cells and enhanced reactive oxygen species (ROS) formation and persistent intracellular curcumin accumulation, thus inhibiting the migration and invasion of melanoma cells more than that observed for free curcumin. Likewise, the same authors showed that with in vivo experiments, curcumin nanoemulsions inhibited the incidence of melanoma and the growth of lung metastatic development.

Similarly, Guant et al. [124] reported that curcumin nanoemulsions showed a high cellular uptake in PC-3 cells (human prostatic carcinoma cell line), showing higher cytotoxicity than unencapsulated curcumin. Machado et al. [125] also showed that the use of curcumin-loaded nanoemulsions in treatment for cancer based on the photosensitization of tumor cells showed a high phototoxic impact, reducing the in vitro cell proliferation and enhancing the ROS levels in MCF-7 and HFF-1 cells.

Resveratrol (3,5,40 -trihydroxy-trans-stilbene) of a lypophilic nature (log *P*_W_^OCT^ = 3.1 [126]) is a natural phytoalexin present in numerous vegetables and fruits (e.g., cranberries, blueberries, grapes, and peanuts). Resveratrol has been reported to induce phase II detoxifying enzymes by activating NRF2 signaling in various human tumor cell lines [72]. However, similarly to curcumin, its therapeutic success has been hindered by its poor bioavailability (is rapidly degradable in high-pH solutions [127] and shows a rapid metabolism [72].

The poor bioavailability of resveratrol is influenced not only by its elevated first-pass metabolism but also by the high degree to which resveratrol attaches to proteins. In the same way, its shelf life and bioavailability (intestinal absorption and cellular uptake) may be enhanced by encapsulating it in a nanoemulsified system. Nanoemulsions protect resveratrol against chemical degradation and avoid isomerization to the less active cis-form [126,128].

For example, Sessa et al. [128] reported that resveratrol encapsulated into nanoemulsions enhanced the resveratrol distribution through cell monolayers, preventing its degradation and providing prolonged release. Resveratrol persisted as encapsulated during in vitro digestion, avoiding metabolization in the gastrointestinal tract, reaching the colon in the active form to be absorbed through the intestinal wall. Lu-Yang et al. [129] also highlighted the high anti-inflammation activity and cytotoxicity of resveratrol nanoemulsions against MCF-7 tumor cells.

Other polyphenol-based NRF2 activators of interest reported to be encapsulated in nanoemulsions to improve their bioavailability are some flavonoids [130]. Aditya et al. [131] reported that the highest bioaccessibility of quercetin in a simulated intestinal environment was obtained when it was encapsulated in a nanoemulsion (~60%) compared to when it was encapsulated in solid lipid nanoparticles (~35%) or when it was unencapsulated (~7%). Epigallocatechin-3-gallate encapsulated in an oil/water nanoemulsion also showed a considerable increase in its in vitro anticancer activity, its bioaccessibility (~2.78-fold), and its intestinal permeability compared to the results obtained for its unencapsulated form [132].

## 5. Challenges of Nano-Sized Drug Delivery Systems

The use of NDDSs has significant potential for improving the diagnosis and treatment of cancer. NDDSs meet the vital needs of specific drug delivery systems to the tumoral target site and achieve high treatment efficiency with a low side effect profile. To provide bench-to-bedside transfer of NDDSs for cancer therapy, several challenges must be overcome. Issues related to large-scale manufacturing, safety, cost, and regulatory requirements should be considered for the clinical development of NDDSs [74,133].

Large-scale production of NDDS is problematic due to their complex structure. Providing batch-to-batch reproducibility in terms of critical NDDS characteristics (particle size, zeta potential, encapsulation efficiency, stability, etc.) is essential for their entrance to the pharmaceutical market. Otherwise, the in vivo pharmacokinetic and pharmacodynamic behaviors may change dramatically. This risk is further increased for complex NDDSs that are modified by the addition of specific ligands, coating materials, different polymers, etc.

Before in vivo preclinical studies, the biocompatibility and safety of NDDSs should be evaluated by nanotoxicology studies on in vitro (cell culture methods), ex vivo, and animal models. There is a need for clear regulatory/safety guidelines and progress is continuing in this regard. Briefly, ensuring the quality assurance of NDDS is necessary but it requires considerable amounts of money and time. Despite all the challenges presented here, the therapy benefits offered by nanoformulations are revolutionary. Considering the length of time from the discovery of a new drug molecule to its launch, it is predicted that the market share of nanoformulations will gradually increase.

## 6. Conclusions

The role of the NRF2 pathway in various cancer types has been demonstrated in clinical studies. Recent developments regarding NRF2 modulators have centered on approaches for cancer therapy and revealed promising outcomes. The major challenges to improving the clinical application of NRF2 modulators include their poor solubility, low bioavailability, instability, and toxicity. NDDSs, such as liposomes, polymeric nanoparticles, micelles, and nanoemulsions, are promising systems for the amelioration of these challenges and clinical translation of NRF2 modulators.

## Figures and Tables

**Table 1:** Studies addressing NRF2 inhibitor-loaded NDDSs for cancer treatment.

Drug Delivery System	Encapsulated Cargo	Formulation Composition	Size (nm)	In Vitro/In Vivo Results	Ref.
Liposome	Plumbagin	soybean PC: chol: mPEG2000-DSPE	115 ± 5	Pharmacokinetic studies on C57BL/6J mice bearing B16F1 melanoma revealed improved AUC and MRT after the i.v. bolus administration (6 mg/kg) of pegylated plumbagin liposomes compared to free plumbagin. Liposomes exhibited better antitumor efficacy and no signs of normal tissue toxicity.	[46]
Liposome	Plumbagin	HPC:DSPE-PEG: chol:chol-PEG- maleimide (60:6:34:0.5)	113 ± 2	Transferrin-bearing liposomes entrapping plumbagin improved the antiproliferative and apoptosis efficacy of cancer cells compared with the drug solution. Upon IV administration to mice bearing tumors, complete tumor eradication for 10% of B16-F10 tumors was provided.	[47]
Liposome	Plumbagin	DPPC DSPC	~100	Combinations of hyperthermia and temperature-sensitive liposomes encapsulating PLB were found to be useful in terms of targeted drug delivery to treat melanoma B16F1 in mice. (IV)	[48]
Liposome	Plumbagin Genistein	l-α-PC mPEG2000-DPPE	~100	The growth of prostate cancer cells (PC-3 rather than LNCaP cells) was synergistically inhibited via plumbagin- and genistein-loaded liposomes (40 to 130 nm). In xenografted tumors, a decrease in the population of proliferative cells and blood vessels was demonstrated upon liposome injection.	[49]
Liposome	Apigenin 5-Fluorouracil	DSPC	105 ± 0.4	A dual drug-loaded liposomal formulation was found to be more cytotoxic against human colon carcinoma cells (HCT-15 and HT-29) compared to free and combinatorial free drugs. This was attributed to a significant upregulation of pAMPK and reversal of the Warburg effect. Preclinical tests in a nude mice tumor xenograft model revealed greater antitumoral activity via the passive targeting of liposomes.	[50]

PC: phosphatidylcholine; Chol: cholesterol; DSPE: 1,2-distearoyl-sn-glycero-3-phosphoethanolamine; HPC: hydrogenated phosphatidylcholine; DSPC: 1,2-distearoyl–sn–glycero-3-phosphocholine; DPPC: dipalmitoyl phosphatidylcholine; DPPE: 1,2-dipalmitoyl-sn-glycero-3-phosphoethanolamine; AUC: area under the curve; and MRT: mean residence time.
